# Effects of Maternal Supplementation with an Injectable Trace Mineral Containing Copper, Manganese, Zinc, and Selenium on Subsequent Steer Finishing Phase Performance and Carcass Characteristics

**DOI:** 10.3390/ani10122226

**Published:** 2020-11-27

**Authors:** Taoqi Shao, Rebecca S. Brattain, Daniel W. Shike

**Affiliations:** Department of Animal Sciences, University of Illinois, Urbana, IL 61801, USA; taoqis2@illinois.edu (T.S.); becca.brattain@kws.com (R.S.B.)

**Keywords:** beef cattle, finishing performance, maternal supplementation, trace minerals

## Abstract

**Simple Summary:**

The persistent effects of maternal nutrition on subsequent offspring health and performance have drawn great attention in both the livestock industry and human health field in recent years. Trace minerals play very important roles in nutrition and regulate many critical biological processes. Therefore, trace mineral status of the dam has the potential to influence early growth and development of the fetus, which leads to long-lasting effects on animal health and growth performance. This study demonstrated that maternal supplementation of trace minerals increased the percentage of carcasses graded as USDA Choice or greater, but maternal trace mineral injections had limited effects on finishing phase growth performance and other carcass characteristics of the offspring. Stakeholders of the cow/calf and feedlot operations should consider these results as they make decisions on maternal trace mineral administrations.

**Abstract:**

The objective of this study was to investigate effects of maternal supplementation with an injectable trace mineral (Cu, Mn, Zn, and Se) on subsequent steer performance during the finishing phase. Seventy-six Angus cross steers (initial body weight 249 ± 41.5 kg) from dams administered either an injectable trace mineral (TM; Multimin 90) or sterilized physiological saline (CON) during prepartum stage were used. Individual feed intake during the finishing phase were recorded with GrowSafe feed bunks. Blood and liver biopsy samples were collected to evaluate trace mineral status. Steers were slaughtered at 413 ± 26 days of age and carcass data were obtained at a commercial abattoir. Growth performance or mineral status of the steers during the finishing phase was not affected (*p* ≥ 0.14) by maternal treatments. Carcass characteristics were not different (*p* ≥ 0.18), except steers from TM dams had greater (*p* = 0.05) percentage of carcasses graded as Choice or greater. In conclusion, maternal supplementation of an injectable trace mineral increased the percentage of carcasses graded as Choice or greater, other than that, maternal supplementation had limited influence on finishing phase growth performance, trace mineral status, or carcass characteristics of the subsequent steer progeny.

## 1. Introduction

For grazing beef cows, supplementation of minerals is needed for optimal reproduction, immune function, lactation, and growth [1,2,3,4]. Trace minerals are present in very low concentrations in the body, but play very important roles in nutrition and regulate many of the critical biological processes. The common practices of supplementing trace minerals to grazing beef cattle include free-choice mineral mixes, trace mineral-fortified salt blocks, and trace mineral-fortified energy/protein supplements [5]. However, voluntary intake fluctuations of the animals are difficult to manage for these common methods of supplementation. An injectable trace mineral provides producers opportunity to deliver specific amounts individually without comprehensive interactions within the gastrointestinal tract [6,7]. For calves, on the other hand, studies indicated that postnatal trace mineral injections at anticipated periods of stress could have beneficial effects on the immune system [8,9] and could reduce morbidity and mortality in early life [10,11]. However, the effects of prenatal/maternal trace mineral injections on offspring growth performance are not fully understood yet.

Fetal programming is the response to a specific environmental challenge during critical periods of fetal growth and development that leads to persistent effects on animal health and growth [12]. Early gestation (first half of gestation) is critical to have maximal placental growth, differentiation, vascularization, and organogenesis [13]; while the last 2 months of gestation accounts for 75% of fetal growth [14]. In ruminants, the supply of minerals for the fetus is completely dependent on the dam [15]. Therefore, it is important to provide minerals in adequate amounts for the pregnant dam to achieve the development of the embryo and fetus throughout the gestation. Recent experiments conducted in ruminant animals have demonstrated that maternal supplementation of trace minerals modifies metabolism [16], body composition [16,17], mineral status [4,18,19], and immune function [20] of the subsequent progeny during the neonatal stage. However, the effects of maternal supplementation of trace minerals on finishing phase performance of the offspring are minimally studied. Therefore, the objective of this study was to investigate effects of maternal supplementation with an injectable trace mineral (Cu, Mn, Zn, and Se) on subsequent steer performance during the finishing phase. We hypothesized that maternal trace mineral supplementation during gestation would improve finishing phase performance and carcass characteristics of the steer progeny.

## 2. Materials and Methods 

Experiment animals were managed according to the guidelines recommended in the Guide for the Care and Use of Agricultural Animal in Agricultural Research and Teaching (Federation of Animal Science Societies, 2010). All experimental procedures were approved by the Institutional Animal Care and Use Committee of the University of Illinois (IACUC #16046).

### 2.1. Experiment Design, Animals, and Management

Seventy-six Angus cross steers were utilized for the current study. The management of the steer calves and dams during the preweaning phase were previously reported by Stokes et al. [4]. In brief, steers were born from heifer dams that received six subcutaneous injections of either trace mineral (TM; Multimin 90; Multimin USA, Fort Collins, CO, USA) or sterilized physiological saline (CON). Prior to artificial insemination (AI), the first three injections were applied at 221, 319, and 401 ± 22 days of age (209, 111, and 29 days prior to AI, respectively). After AI, the other three injections were maintained and applied on 205, 114, and 44 ± 26 days prepartum. The Multimin 90 contained 15 mg/mL of copper, 10 mg/mL of manganese, 60 mg/mL of zinc, and 5 mg/mL of selenium. The administration rate was 1 mL/45 kg body weight (BW) at 221 and 319 ± 22 days of age, and adjusted to 1 mL/68 kg BW for the subsequent injections according to the manufacture’s recommendation. All steers were weaned at 159 ± 26 days of age and transported on the following day approximately 350 km to the Beef Cattle and Sheep Field Laboratory in Urbana, IL, USA.

Steers were housed in barns with slatted concrete floors covered by interlocking rubber matting. Pens were constructed with galvanized steel tubing to 14.64 m × 4.88 m in dimension. A subset (*n* = 20) of the steers were used in an immune challenge study, which was described by Stokes et al. [21]. Upon arrival (day −2), the remainder of the steers (*n* = 56) were given access to a free choice of grass hay and provided with a receiving ration in concrete bunks for 1 week. After the first week of receiving phase (day −5), all 76 steers were managed and fed a common adaptation diet for 5 weeks. The receiving and the adaptation diets were reported previously by Stokes et al. [21]. Steers were adapted to GrowSafe feed bunks (GrowSafe System Ltd., Airdrie, AB, Canada) for individual daily feed intake measurement during the last three weeks of the adaptation phase. Thereafter, the full BW of the steers was measured for two consecutive days for the initial (days −1 and 0) and the end of finishing phase (days 186 and 187) of the finishing phase. The diet and nutrient composition during the finishing phase are presented in Table 1. Based on the initial BW on the first day (day −1), steers were blocked by BW (249 ± 42 kg) into 2 groups (heavy and light) and assigned into 6 pens with 12 or 13 steers per pen. Within each block, previous dam treatments and lipopolysaccharide challenge treatments [21] were stratified into 3 pens. Besides the initial and final two-day weighing, steers were weighed on days 42, 84, 102, and 137 to monitor growth performance. Steers were implanted on day 0 with Component TE-IS (16 mg estradiol and 80 mg trenbolone acetate; Elanco Animal Health, Greenfield, IN, USA) and d 102 with Component TE-S (120 mg trenbolone acetate, 24 mg estradiol USP, and 29 mg tylosin tartrate; Elanco Animal Health, Greenfield, IN, USA). Steers were fed 200 mg/steer/day of Optaflexx 45 (99 g/kg, Elanco Animal Health, Greenfield, IN, USA) for the last 28 d before slaughter. On day 187, steers (average 413 ± 26 days of age) were transported approximately 310 km to a commercial abattoir and slaughtered humanely. On the day of slaughter, hot carcass weight (HCW) was measured; while after a 24 h carcass chill, carcass characteristics were determined by United States Department of Agriculture (USDA) camera system.

One steer from CON was removed from the trial because of chronic bloat. All data from this steer was included in analysis until the date of removal.

### 2.2. Sampling and Analytical Procedures

Sixteen days prior to slaughter, blood (*n* = 18) and liver biopsies (*n* = 16) were collected for trace mineral determination. Blood samples were collected from the jugular vein of steers by using trace element serum vacuum tubes (6.0 mL; Becton, Dickinson, and Co., Franklin Lakes, NJ, USA). Blood samples were given two hours for clotting under room temperature and centrifuged at 1300× *g* for 20 min at 4 °C. Liver biopsies were collected and stored with the methods described by Stokes et al. [4]. Blood and liver samples were shipped to Michigan State University Diagnostic Center for Population and Animal Health (Lansing, MI, USA). Biopsies were dried overnight in a 75 °C oven to determine the dry matter fraction and calculate the dried tissue mass. The dried biopsies were digested in nitric acid in a 95 °C oven for four hours. The digested samples were diluted with water to 100× the dried tissue mass. Concentrations of Cu, Mn, Zn, and Se were measured using an Agilent 7500ce Inductively Coupled Plasma Mass Spectrometer (Agilent Technologies Inc., Santa Clara, CA, USA) via procedures reported by Wahlen et al. [23].

Feed samples for nutrient composition analysis were collected every four weeks during the finishing phase and stored at −20 °C until further processing. Samples were composited and dried at 55 °C for 3 days, then ground through a 1 mm screen using a Wiley mill (Arthur H. Thomas, Philadelphia, PA, USA). Feed samples were analyzed for dry matter (DM), crude protein (CP; TruMac; LECO Corporation, St. Joseph, MI, USA), crude fat (Ankom XT10 Fat extractor, Ankom Technology, Wayne, NY, USA), neutral detergent fiber (NDF), and acid detergent fiber (ADF; Ankom 200 Fiber Analyzer, Ankom Technology, Macedon, NY, USA).

### 2.3. Statistical Analysis

Individual animal was considered the experimental unit for all response variables. Data were analyzed using the MIXED procedure of SAS (SAS Inst. Inc., Cary, NC, USA). Repeated measure was used for the analysis of BW, which used unstructured covariance structure based on the Akaike information criterion with the smallest AIC, AICC, and BIC. The model for the analysis of BW included fixed effects of treatment, time, and the interaction of treatment and time, and random effect of pen nested within block, while birthdate and expected progeny differences (EPD) of average daily gain (ADG) were included as covariates. The model for the analysis of dry matter intake (DMI), ADG, and gain-to-feed ratio (G:F) included a fixed effect of treatment and a random effect of pen nested within block, while EPD of ADG as a covariate. The model for HCW, dressing percentage, longissimus muscle (LM) area, 12th rib fat thickness, yield grade (YG), and kidney pelvic heart fat (KPH) included fixed effects of treatment, sire, birthdate, and random effect of pen nested within block. The initial model for marbling score included treatment as the fixed effect and pen nested within block, and marbling EPD and birthdates as the covariates. Pen nested within block and birthdate were removed because of non-significance. Data concerning the distribution of quality grade (QG) and YG were analyzed using the GLIMMIX procedure of SAS. The model for QG and YG distribution included the fixed effect of treatment, while marbling EPD and yield grade EPD were included as covariates for QG and YG, respectively. Treatment effects were considered significant at *p* ≤ 0.05 and tendencies at 0.05 < *p* ≤ 0.10.

## 3. Results

### 3.1. Growth Performance

The BW of the steer progeny during the finishing phase are presented in Table 2. There was no treatment × time interaction (*p* = 0.46) or treatment effect (*p* = 0.95) for steer BW during the finishing phase. Feed efficiency related parameters are presented in Table 3. In the finishing phase, there were no differences (*p* ≥ 0.20) in ADG, DMI, or G:F. With similar initial BW and ADG for the finishing phase, the final BW of the steers was not different.

### 3.2. Trace Mineral Status

Trace mineral status in serum and liver prior to slaughter are presented in Table 4. There were no differences (*p* ≥ 0.14) in serum or hepatic Cu, Mn, Se, or Zn concentration between different maternal treatments.

### 3.3. Carcass Characteristics

The results regarding carcass characteristics are presented in Table 5. Steers from TM dams have greater (*p* = 0.05) percentage of carcasses graded as Choice compared to the steers from CON dams. However, there was no difference (*p* = 0.18) on the average marbling score.

No differences (*p* ≥ 0.40) were detected on HCW, dressing percentage, LM area, 12th rib fat thickness, or KPH percentage. There was no difference (*p* ≥ 0.32) in carcass YG or the percentage of carcasses graded as YG 1, 2, 3, or 4.

## 4. Discussion

### 4.1. Growth Performance

In the current study, the growth performance of the steer progeny during the finishing phase was not affected by maternal trace mineral injections. Marques et al. [19] reported that weaning BW of the calves born from dams supplemented with an organic complexed source of Cu, Mn, Co, and Zn during the last trimester of gestation was greater compared to those from non-supplemented dams, and this improvement was maintained until the end of the finishing phase. However, the weaning BW of the steers in the current study was similar between CON and TM [4]. With similar initial BW and ADG for the finishing phase, the final BW of the steers was not different. Similarly, Marques et al. [19] reported that the finishing phase ADG of the calves was not affected by maternal trace mineral supplementation. The difference in final BW of the calves came from weaning BW, which according to the authors could be due to programming effects on the immune function by supplementing Zn, Cu, Mn, and Co to late gestation dams [19]. However, according to a lipopolysaccharide challenge conducted by Stokes et al. [21], injectable maternal supplementation of Zn, Cu, Mn, and Se to pregnant dams had minimal effects on the offspring immune response following an immune challenge during the feedlot receiving period. Having different offspring responses could be mainly due to different amount and type of trace minerals supplemented to dams. However, further investigations need to be conducted and repeated as limited studies have been focused on the effects of maternal trace mineral supplementation on offspring performance after weaning.

### 4.2. Trace Mineral Status

Trace mineral concentrations in either the serum or liver of steer progeny during the finishing phase were not affected by maternal supplementation of trace mineral injections. Blood measures on trace minerals may provide limited information on the trace mineral status as circulating trace minerals are regulated by homeostatic mechanisms until depletion of the hepatic reserves [24], while liver is the ideal organ for the analysis of trace mineral metabolism as other tissues do not consistently reflect the actual trace mineral status [25]. The studies that have investigated maternal trace mineral supplementation mainly focused on the neonatal period of the offspring. Stokes et al. [4] reported that the concentrations of hepatic Cu and Se were increased in TM calves at birth, while concentrations of trace minerals in plasma were not different. Under the same study, hepatic trace mineral concentrations were not different at weaning (average 147 days of age) regardless of maternal treatment [4]. Given the hepatic concentrations of trace minerals were similar at weaning, it is not surprising that the trace mineral status during the finishing phase was not different in the current study. In dairy calves born from dams supplemented with Se-yeast during the last 8 weeks of gestation, the concentrations of whole blood Se and serum Se for the first 2 weeks of age were greater compared to the control groups [18]. In placental cotyledons from dams supplemented with sulfate sources of trace minerals and organic complexed source of trace minerals, Co concentrations were increased compared with non-supplemented ones [19]. However, limited data is available on the trace mineral status during later life, like the finishing phase. The current study indicates that the maternal supplementation had limited impacts on hepatic concentrations of trace minerals during the finishing phase.

### 4.3. Carcass Characteristics

Increased intramuscular adipogenesis during the fetal stage leads to a greater number of intramuscular adipocytes, which provide sites for marbling formation during the finishing phase [26]. Limited studies have been done in ruminant animals investigating effects of maternal trace mineral supplementation on adipogenesis of the subsequent progeny. In vitro, Cu increases the differentiation of mesenchymal stem cells into adipogenic lineage [27]. Selenium has been used as a part of the adipogenic differentiation media of pigs and rats [28,29]. Hassan et al. [30] reported a proadipogenic role of Se during the chicken embryonic development. In addition, maternal Se supplementation was reported to be able to increase adiposity in female lamb progeny [16]. Huang et al. [31] reported that zinc-finger protein 423 regulates adipogenesis differentiation in bovine muscle and may be able to enhance intramuscular adipogenesis and marbling. These data indicate trace minerals, such as Cu, Se, and Zn, have potential to promote adipogenesis. Although marbling score was not significantly impacted by treatment, there was a 34-point numerical difference between TM and CON, which translated into significantly different quality grade distributions. For the calves utilized in the current study, concentrations of hepatic Cu and Se at birth (5 ± 3 days postpartum) were greater in TM compared to CON [4], which could have led to greater adipogenesis in the early stage of the TM calves and contributed to the greater proportion of carcass graded as Choice or greater. However, mechanisms relating to maternal trace mineral supplementation on adipogenesis of the offspring need further investigation, especially in ruminant animals.

## 5. Conclusions

Maternal supplementation of an injectable trace mineral containing copper, manganese, zinc, and selenium had limited effects on finishing phase growth performance and trace mineral status. Steers from TM dams had an increased percentage of carcasses graded as Choice or greater. However, supplementing an injectable trace mineral to gestating beef cows had minimal influence on other carcass characteristics of the subsequent steer progeny. Future research is needed to determine the effects of maternal trace mineral supplementation on the early adipogenesis of the fetus, which might further impact the marbling formation during the finishing phase.

## Figures and Tables

**Table 1:** Diet and nutrient composition during fishing phase.

Item	Inclusion, % Dry Matter (DM)		
	Day 0 to 76	Day 77 to 90	Day 91 to d 187
Ingredient, %			
High moisture corn	30	20	10
Dry-rolled corn	30	40	50
Distillers grains	20	20	20
Corn silage	10	10	10
Supplement ^1^	10	10	10
Chemical Analysis, % DM			
Dry matter	68.4	70.0	70.7
Crude protein	14.6	14.7	14.8
Neutral detergent fiber	16.8	16.8	16.8
Acid detergent fiber	7.2	5.6	7.1
Ether extract	5.6	5.6	5.6
NEm ^2^, Mcal/kg	2.11	2.10	2.10
NEg ^2^, Mcal/kg	1.45	1.44	1.43

^1^ Supplement contained 73.4% ground corn, 17.8% limestone, 6.7% urea, 1.0% trace mineral premix, 0.17% Rumensin 90, 0.11% Tylan 40, and 0.84% fat. Trace mineral premix contained 8.5% Ca, 5% Mg, 7.6% K, 6.7% Cl, 10% S, 0.5% Cu, 2% Fe, 3% Mn, 3% Zn, 278 mg/kg Co, 250 mg/kg I, 150 mg/kg Se, 2205 KIU/kg Vit A, 662.5 KIU/kg Vit D, 22,047.5 IU/kg Vit E. Tylan 40 (Elanco Animal Health, Greenfield, IN, USA); ^2^ Calculated net energy for maintenance (NEm) and net energy for gain (NEg) values from National Academies of Sciences, Engineering, and Medicine [22].

**Table 2:** Effects of maternal supplementation of trace minerals on the subsequent steer body weight (BW) during the finishing phase.

Item	CON	TM	SEM	*p*-Value		
				Trt	Time	Trt × Time
**Body Weight, kg**				0.95	<0.01	0.46
Initial ^1^	247	250	3.5			
day 42	323	323	4.8			
day 84	408	409	5.3			
day 102	445	445	5.8			
day 137	516	511	6.8			
day 187	593	592	8.5			

^1^ Body weight at 225 ± 26 days of age; measured two consecutive days at the initiation of the finishing phase. CON = maternal injections of sterilized physiological saline during prebreeding and gestation; TM = maternal injections of Multimin 90 during prebreeding and gestation; SEM = standard error of the mean; Trt = treatment effect; Trt × Time = interaction between treatment and time.

**Table 3:** Effects of maternal supplementation of trace minerals on the finishing performance of subsequent steer progeny during the finishing phase.

Item	CON	TM	SEM	*p*-Value
ADG, kg/d	1.87	1.82	0.041	0.20
DMI, kg/d	10.1	9.7	0.27	0.22
G:F	0.185	0.189	0.0052	0.44

CON = maternal injections of sterilized physiological saline during prebreeding and gestation; TM = maternal injections of Multimin 90 during prebreeding and gestation; ADG = average daily gain; DMI = dry matter intake; G:F = gain to feed ratio; SEM = standard error of the mean.

**Table 4:** Effects of maternal supplementation of trace minerals on serum and hepatic trace mineral concentrations of steer progeny during the finishing phase.

Item	CON	TM	SEM	*p*-Value
Serum, μg/mL				
Cu	0.868	0.769	0.0622	0.14
Mn	1.773	1.945	0.159	0.3
Zn	1.209	1.285	0.1052	0.48
Se	82.5	79.3	5.07	0.54
Liver, μg/g DM				
Cu	204.9	188.3	40.13	0.69
Mn	8.29	7.92	1.134	0.75
Zn	99.7	97.9	13.46	0.9
Se	1.35	1.28	0.185	0.73

CON = maternal injections of sterilized physiological saline during prebreeding and gestation; TM = maternal injections of Multimin 90 during prebreeding and gestation; SEM = standard error of the mean.

**Table 5:** Effects of maternal supplementation of trace minerals on carcass characteristics of subsequent steer progeny.

Items	CON	TM	SEM	*p*-Value
No. of steers	34	39		
Yield				
HCW, kg	358	353	8	0.47
Dressing percentage, %	60.8	60.5	0.36	0.4
LM area, cm^2^	12.8	12.67	0.251	0.59
12th rib fat thickness, cm	1.45	1.53	0.101	0.44
KPH, %	2.1	2.1	0.04	0.69
Average YG	3.39	3.42	0.154	0.82
YG 2, %	23.5	34.4	-	0.36
YG 3, %	49.5	37.5	-	0.32
YG 4, %	13.2	16.4	-	0.7
Quality				
Average marbling score ^1^	462	496	24.3	0.18
Select, %	28.8	9.9	-	0.05
Low choice or greater, %	71.2	90.1	-	0.05
Average choice or greater, %	31.1	40	-	0.44
Prime, %	0	10	-	0.97

^1^ Marbling score: <400 = Select; 400 = Low Choice; 500 = Average Choice; 700 = Prime. CON = sterilized physiological saline injections; TM = Multimin 90 injections; HCW = hot carcass weight; LM area = longissimus muscle area; KPH = kidney pelvic heart fat; YG = yield grade; CON = maternal injections of sterilized physiological saline during prebreeding and gestation; TM = maternal injections of Multimin 90 during prebreeding and gestation; SEM = standard error of the mean.
